# An experimental phylogeny to benchmark ancestral sequence reconstruction

**DOI:** 10.1038/ncomms12847

**Published:** 2016-09-15

**Authors:** Ryan N. Randall, Caelan E. Radford, Kelsey A. Roof, Divya K. Natarajan, Eric A. Gaucher

**Affiliations:** 1 School of Biological Sciences, Georgia Institute of Technology, Atlanta, Georgia 30332, USA; 2 Institute for Bioengineering and Biosciences, Georgia Institute of Technology, Atlanta, Georgia 30332, USA

## Abstract

Ancestral sequence reconstruction (ASR) is a still-burgeoning method that has revealed many key mechanisms of molecular evolution. One criticism of the approach is an inability to validate its algorithms within a biological context as opposed to a computer simulation. Here we build an experimental phylogeny using the gene of a single red fluorescent protein to address this criticism. The evolved phylogeny consists of 19 operational taxonomic units (leaves) and 17 ancestral bifurcations (nodes) that display a wide variety of fluorescent phenotypes. The 19 leaves then serve as ‘modern' sequences that we subject to ASR analyses using various algorithms and to benchmark against the known ancestral genotypes and ancestral phenotypes. We confirm computer simulations that show all algorithms infer ancient sequences with high accuracy, yet we also reveal wide variation in the phenotypes encoded by incorrectly inferred sequences. Specifically, Bayesian methods incorporating rate variation significantly outperform the maximum parsimony criterion in phenotypic accuracy. Subsampling of extant sequences had minor effect on the inference of ancestral sequences.

## Abstract (web summary)

Methods for ancestral sequence reconstruction are currently tested with computer simulations, since true biological phylogenies are unknown. Here, Randall *et al.* build an experimental phylogeny to benchmark the performance of alternate ancestral sequence reconstruction algorithms in inferring ancestral genotypes and phenotypes.

## Graphical abstract

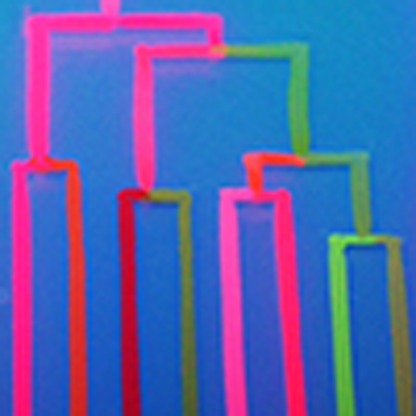

Ancestral sequence reconstruction (ASR) is the process of analyzing modern sequences within an evolutionary/phylogenetic context to infer the ancestral sequences at particular nodes of a tree^1^. These ancient sequences are most often then synthesized, recombinantly expressed in laboratory microorganisms or cell lines, and then characterized to reveal the ancient properties of the extinct biomolecules^2^,^3^,^4^,^5^,^6^. This process has produced tremendous insights into the mechanisms of molecular adaptation and functional divergence^7^. Despite such insights, a major criticism of ASR is the general inability to benchmark accuracy of the implemented algorithms. It is difficult to benchmark ASR for many reasons. Notably, genetic material is not preserved in fossils on a long enough time scale to satisfy most ASR studies (many millions to billions of years ago), and it is not yet physically possible to travel back in time to collect samples.

To overcome these limitations, we exploited an under-utilized yet effective procedure to develop a phylogeny in the laboratory^8^. The benefits of the procedure are at least twofold: (1) we can accelerate the process of evolution that generates the vertical inheritance of genetic information necessary for the functional divergence of encoded phenotypes and (2) we have a known record of the ancestral genotypes and phenotypes throughout the experimental phylogeny. The goal of the phylogeny is thus to create an opportunity to evolve sequences within a controlled framework that adds biological reality given practical limitations. We elected to build the phylogeny using a single monomeric red fluorescent protein (FP), since it is known that FP colour phenotypes are readily modified by a tractable number of amino acid replacements^9^,^10^. The experimental phylogeny then provides us with an opportunity to benchmark the performance of algorithms that infer ancient sequences. In particular, we were interested in determining the accuracy of algorithms when inferring ancestral phenotypes since computer simulations have shown that these algorithms infer ancient genotypes with reasonably high accuracy^11^,^12^,^13^. Our benchmarking exercise focused on Bayesian versus maximum parsimony (MP) algorithms, the effect of rate variation when modelled as a discrete gamma distribution^14^, subsamples of taxa to infer ancestral sequences, and species-tree-aware versus unaware approaches within the Bayesian framework^15^,^16^.

Our study confirms that all ASR algorithms correctly infer the vast majority of residues in ancestral sequences. Yet, these algorithms differ in the amino acid identities of the small number of sites that are incorrectly inferred. Here we demonstrate that these incorrectly inferred residues can indeed influence the protein phenotypes of the encoded ancestral sequences and that various parameters incorporated into evolutionary models affect these incorrectly inferred sites.

## Results

### Evolving the experimental phylogeny

**Figure 1:**
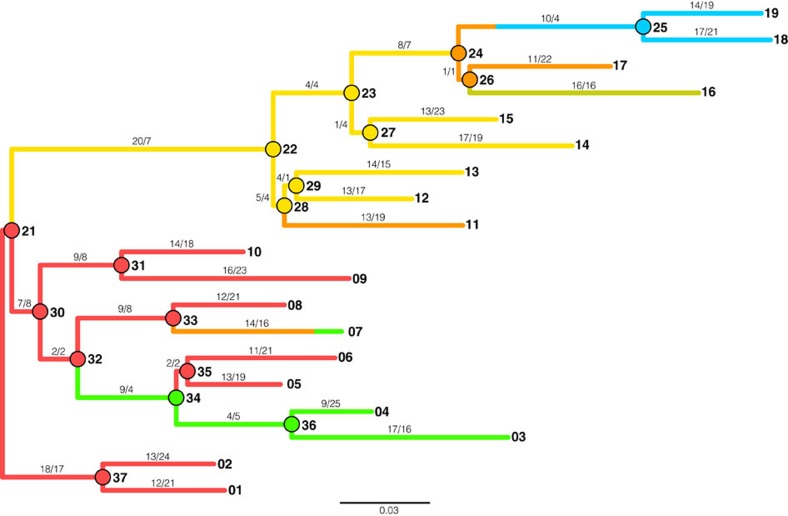
Phylogram of the experimental phylogeny initiated from a single red FP gene. Scale bar represents amino acid replacements per site per unit evolutionary time. The colour of each branch reflects the colour-class phenotype (emission) of the node protein for internal branches or the leaf protein for tip branches (except for the branch connecting node 33 to leaf 7 that transitions through an orange intermediate). Nodes and tips are numbered for reference. Nonsynonymous and synonymous substitutions are shown along each branch, respectively. The experiment began near node 21 with a single red FP gene and proceeded by random-mutagenesis PCR.

We built the FP phylogeny from a single gene using random mutagenesis PCR (Fig. 1). Each round of PCR produced numerous variants, or descendants, of which only one was retained for the next round of random mutagenesis, unless a bifurcation was being incorporated into the tree, in which case two variants would be allowed to progress (Supplementary Fig. 1). To best create biological context, the branch lengths, the number of synonymous and nonsynonymous substitutions, base frequencies, and phenotypic diversity followed that of natural FP sequences^17^. In total, the phylogeny contains 19 operational taxonomic units (leaves of the tree) that serve as ‘modern' sequences and 17 ancestral bifurcations (nodes of the tree) that serve as true or known ‘ancient' sequences. The phylogeny contains a total of 833 mutations (461 synonymous and 372 nonsynonymous), with a small percentage of these experiencing homoplasy, but no insertion/deletion events (Supplementary Table 1). Transitions were more abundant than transversions, 64% versus 36%, respectively. Most branches were evolved under purifying selection except when selecting for modifications in the phenotypic emission properties of the FPs. Colour properties of the evolved proteins included variations of red, orange, yellow, green and blue (Supplementary Fig. 2). The order and distribution of colour emission phenotypes for FP proteins were mapped onto the phylogeny (Supplementary Fig. 3).

### Inferring ancient sequences

**Figure 2:**
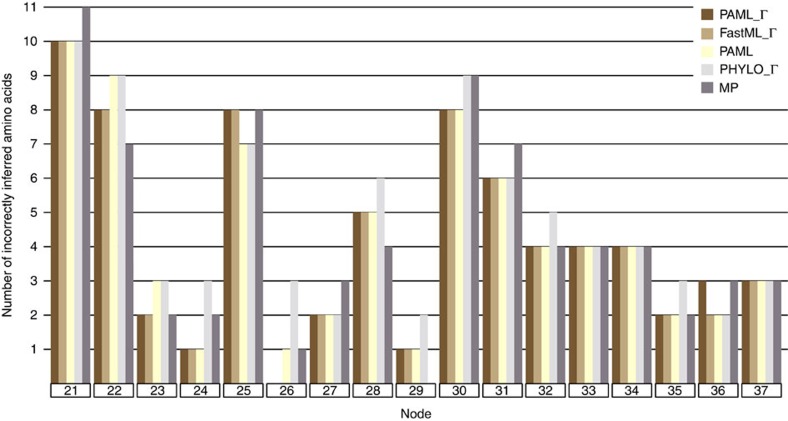
Number of incorrectly inferred amino acid sites for each node of the phylogeny. The 19 leaf sequences from Fig. 1 were subjected to ASR analyses using Bayesian (PAML, FastML, PhyloBayes) with or without rate variation modelled as a gamma distribution (Г), as well as parsimony (MP). The inferred sequences were then compared to the true ancestral sequences from the 17 ancestral nodes in Fig. 1. Dark brown bars are PAML with a gamma distribution, light brown bars are FastML with a gamma distribution, yellow bars are PAML without gamma, light grey bars are PhyloBayes with a gamma distribution, and dark grey bars are maximum parsimony. Colour code is irrespective of FP colour emission phenotype.

The 19 leaf-sequences were collected and subjected to ASR analyses. The sequences were analysed using MP and Bayesian algorithms, and for the Bayesian approach^18^,^19^, we analysed the effects of incorporating rate variation (gamma distribution [Г] versus no gamma distribution, or rate heterogeneity versus rate homogeneity) and the effect of accounting for possible gene duplication, horizontal transfer, or gene loss events (so-called species-tree-aware trees, as implemented in PhyloBayes)^16^. Figure 2 shows the results from five different ASR analyses across all nodes of the phylogeny as a function of the number of incorrectly inferred ancestral amino acids. This figure shows the expected pattern that all ASR procedures perform well for more derived nodes, while all procedures perform worse for more basal nodes. In terms of raw percentage of correctly inferred residues, most procedures recapitulated reality (Supplementary Table 2). The Bayesian approaches that incorporated rate variation using a species-tree-unaware tree were the most accurate (PAML_Г and FastML_Г, Supplementary Table 3a,c), then Bayesian without rate variation (PAML), followed by MP, and finally by Bayesian with rate variation and species-tree-aware tree using PhyloBayes (PHYLO_Г). PAML and FastML were expected to perform analogously since they are similar implementations of the Bayesian algorithm. Total accuracy for the five procedures ranged between 97.88 and 98.17% (Supplementary Table 2), thus reflecting the general sequence accuracy of ASR algorithms.

### Characterizing the evolved and inferred protein phenotypes

**Figure 3:**
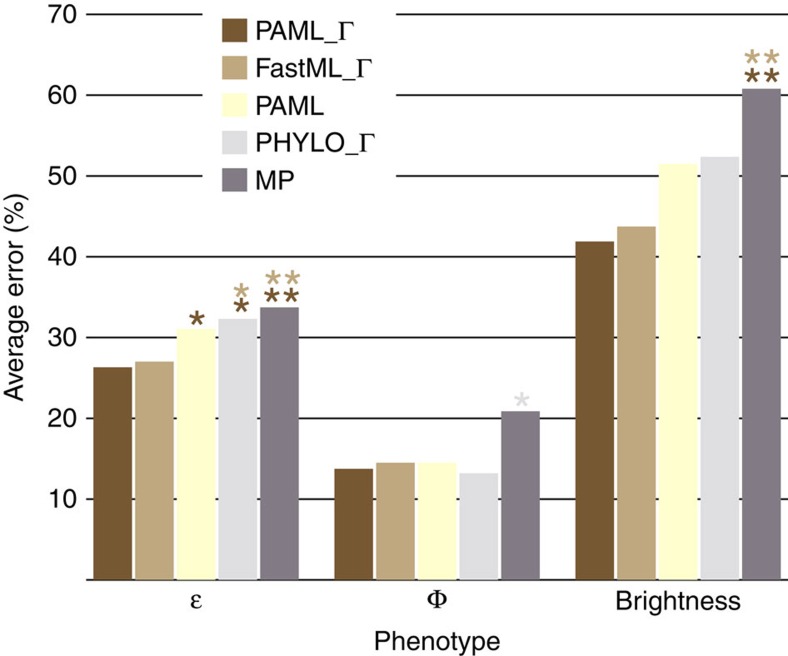
Average phenotypic error across all nodes for the five ASR procedures. Extinction coefficient (ɛ), quantum yield (Φ), and brightness (product of ɛ and Φ) were determined for all incorrectly inferred ancestral FP proteins and compared to the properties of the true ancestral protein at each node and reported as a function of percent error. Dark brown bars are PAML with a gamma distribution, light brown bars are FastML with a gamma distribution, yellow bars are PAML, light grey bars are PhyloBayes with a gamma distribution, and dark grey bars are maximum parsimony. Single and double asterisks represent confidence at 95% and 99% levels, respectively, and are coloured according to the respective procedure that has significantly less error.

Despite the overall sequence accuracies of the five procedures, we questioned whether the phenotypes associated with the incorrectly inferred ancestral sequences are themselves incorrect. We synthesized, expressed and purified each incorrectly inferred ancestral protein at each node of the tree for each procedure to determine whether there was variation in the phenotypes for the incorrect proteins compared with the true ancestral phenotypes. These 34 proteins were phenotypically characterized in terms of their extinction coefficients (ɛ), quantum yield (Φ) and brightness (product of ɛ and Φ) (Supplementary Table 3a,b). Properties of the resurrected ancestral proteins were compared with the true ancestral proteins to determine the percent error in phenotypes. Figure 3 shows that significant variation in phenotypic error exists between the five procedures. PAML_Г and FastML_Г had significantly less error than PAML without rate variation and PHYLO_Г at the 95% confidence interval, and less error than MP at the 99% level when characterizing extinction coefficients. Conversely, PHYLO_Г generated significantly lower error compared with MP for quantum yield at the 95% level, with the other three Bayesian procedures only slightly worse than PHYLO_Г. The brightness phenotype demonstrated that PAML_Г and FastML_Г carried over their significantly lower error compared to MP at the 99% level, and that PAML without rate variation and PHYLO_Г displayed similar amounts of error less than MP. None of the five procedures displayed significant error for emission wavelengths, and as seen in Supplementary Table 3a, little error was displayed for excitation wavelengths.

## Discussion

We have applied brute-force random-mutagenesis and guided-selection to generate an experimental phylogeny of synthetic FPs to recapitulate evolutionary processes that govern natural FPs^17^. This phylogeny contained ample phenotypic diversity analogous to most gene families subjected to ASR studies. Despite best efforts, we anticipate that experimental bias exists within the phylogeny to some degree, but we should not be paralysed since natural molecular systems routinely display bias (for example, G+C, transitions, base composition and so on) and parameter-based models account for such bias. The experimental phylogeny allowed us to verify that ASR often generates correct ancestral phenotypes even when the wrong ancestral sequences have been inferred in our FP system (Supplementary Table 3a,c). We anticipate that such accuracy would hold true for other laboratory-evolved gene families since there is no reason to think that FP evolution involves an extraordinary mechanism *per se*. However, improvements can still be made to these phylogenetic algorithms. In particular, we tested known limitations of these algorithms by purposely invoking homoplasy into the experimental phylogeny. The five procedures performed generally well with reversions, and parallel and convergent amino acid replacements, as long as they occurred along sufficiently long branches (Supplementary Fig. 1). This was not the case for one scenario though. Ancestral node 32 (An32) accumulates an amino acid replacement (Y120C) that causes the colour phenotype to switch from red to green by An34 (Supplementary Fig. 1). The reversion of C120Y then occurs along the short branch giving rise to a red An35. None of the five procedures could account for this mode of homoplasy, thus they all predicted that An34 was red. Notably, this is the only incorrectly inferred protein displaying emission in a separate colour class than the true ancestor. Interestingly, all five procedures predicted sequences that encode the correct colour emission for An35 yet all of these sequences encode the highly incorrect quantum yield carried over from An34 (Supplementary Table 3a). This result demonstrates that protein properties encoded by incorrect sequences can propagate throughout nodes connected by short branches—a caution for ASR studies. Notwithstanding such known difficulties of homoplasy, all five procedures performed admirably despite the phylogeny experiencing dramatic phenotypic changes. For instance, the branch connecting An33 and leaf 7 switched from red to orange to green colour phenotypes, but all five procedures correctly inferred the colour phenotype despite each incorrectly inferring four amino acid sites (Figs 1 and 2, Supplementary Table 3a). Similarly, all five procedures correctly inferred the colour phenotype of An24 despite that only one of the four descendent leaves had the same colour phenotype as the ancestor (and despite that the five procedures incorrectly inferred 1–3 amino acid sites; Figs 1 and 2, Supplementary Table 3a).

Taxon sampling in phylogenetics has been greatly debated over the past 20 years^20^. Central to this debate is whether sub-samplings of taxa lead to inferences of incorrect phylogenies. Computer simulations and large data sets of angiosperms have supported the conclusion that robust taxa samples are superior than smaller subsamples of taxa^20^,^21^, while others have argued against this conclusion^22^,^23^. To address the issue of sub-sampling of taxa in the inference of ancestral sequences (but not the topology itself), we generated two diverse subsamples from our 19 leaf sequences. Our analysis focused on the last common ancestor (most ancient divergence, An21 from Fig. 1) of the phylogeny since this sequence is theoretically the most difficult to correctly infer. One subsample incorporated every other sequence along the continuum of leaf sequences, while the other subsample consisted of closely related groups of leaf sequences (Supplementary Fig. 4). Both of these subsamples utilized half the number of leaf sequences and inferred a sequence for the last common ancestor of the two reduced phylogenies. Each of the two ancestors differed at only a single position when compared with the 10 incorrect residues inferred using the entire 19 sequences with WAG_Г (at An21). The single amino acid position that differed from each subsample analysis experienced sufficient homoplasy throughout the assembly of the experimental phylogeny yet was not associated with phenotypic change. Although these sub-sampling analyses focus on the effects of using half of the leaf sequences to infer the last common ancestor, and showed little effect, additional sub-samplings should be explored to gain additional insights into the effects of subsamples in ASR studies^20^.

Overall, our experimental phylogeny has allowed ASR algorithms to be benchmarked for the first time against biologically encoded sequences. Our analyses focused on amino acid reconstructions since they performed slightly worse than DNA- and codon-based analyses (98.4% and 98.3% of sites correctly inferred, respectively, Supplementary Table 2). These analyses confirm computational predictions regarding the accuracy of ASR but extend our understanding of the algorithms by revealing the phenotypes associated with incorrectly inferred ancestral sequences. All of the tested ASR algorithms and procedures work generally well in terms of capturing the true ancestral phenotype even when the true ancestral genotype is not fully recapitulated. This finding should give the ASR field confidence that ancestral phenotypes are encoded correctly even if some residues are incorrectly inferred—assuming such sites do not drive phenotypes. Our experimental phylogeny has also allowed us to determine nuances of ASR analyses. For instance, incorporating rate variation using a discrete gamma distribution had little effect on the total number of incorrectly inferred amino acids (71 with gamma versus 72 without gamma, Supplementary Table 2), however, the positions of these incorrectly inferred sites differed, and more interestingly, the encoded phenotypes differed substantially in terms of brightness error between the two procedures (42% with gamma versus 51% without gamma, Supplementary Table 3c). Intriguingly, our analyses did not find a strong correlation between the number of incorrectly inferred residues versus the errors in the measured phenotypes. Since more-ancient nodes often contain more incorrectly inferred residues^11^,^13^, this suggests that more-ancient nodes are not necessarily encoding furtherly biased phenotypes. Further, our analyses demonstrated that incorporating a species-tree-aware procedure had the same overall effect as not incorporating rate variation. This is not to say that species-tree-aware procedures inherently mislead, rather, our experimental phylogeny was void of gene loss/gain/duplication so a PhyloBayes analysis would be over-parameterized for our dataset. But our results do suggest that a species-tree-unaware procedure is more appropriate in the absence of gene loss/gain/duplication or lineage sorting. Finally, our analyses demonstrate that Bayesian procedures produce more accurate ancestral phenotypes than the MP criterion, regardless of the Bayesian parameters tested. This is not a general abomination against parsimony, as it performed quite accurately for many nodes in the phylogeny. Rather, if the goal is to generate the most accurate ancestral phenotypes possible, then ASR studies on gene families that have evolved (at least) analogously to our laboratory FP system would benefit most from Bayesian procedures.

We anticipate that providing assurance on the accuracy of ASR will allow the field to move forward in novel ways, such as the synthesis of complete ancestral genomes^24^,^25^,^26^, development of mechanistic models of protein evolution^27^,^28^, contribute to the debate about alternative ancestral sequences^11^,^13^,^18^ and to support synthetic biology approaches that exploit ASR for applied purposes^29^,^30^,^31^.

## Methods

### Evolving the experimental phylogeny

The following two primers were used for PCR mutagenesis: FP Random Forward (5′-CTGGTCGGCCATATGGCGTCTTCTGAAGACGTTATC-3′) and FP Random Reverse (5′-CGGATCCTCGAGCTATTACGCACCGGTAGAGTG-3′). Random mutagenesis of *mRFP1* was performed using the GeneMorph II Random Mutagenesis Kit (Stratagene). Each reaction was performed in 50 μl and consisted of the following: 425–625 ng template plasmid, 0.25 μl forward primer, 0.25 μl reverse primer, 1 μl of 40 mM dNTP stock, 5 μl 10 × Mutazyme II reaction buffer, 1 μl Mutazyme II DNA polymerase. PCR was performed using the following conditions: initial incubation at 95 °C 2 min then 95 °C 30 s, 59 °C 30 s, 72 °C 1 min, repeat × 29, final incubation at 72 °C 10 min. PCR products were purified using Qiagen PCR clean up kit following the manufacturer's protocol. Purified mutagenesis PCR DNA was digested in a 50 μl reaction at 37 °C between 16–48 h and included the following: FP DNA, 1 μl *Xho*I, 1 μl *Nde*I, 5 μl Buffer 4, 0.5 μl BSA. Digested DNA was cleaned up using Qiagen's PCR clean up kit following the manufacture's protocol. The digested and cleaned FP mutant genes were ligated into pET-15b (Novagen) according to the following protocol: 100 ng digested pET-15b Vector, 20 ng digested FP gene, 0.5 μl T4 Ligase, 1–2 μl 10 × T4 Ligase Buffer in a 10–20 μl reaction. Plasmids containing mutated mRFP 1.0 were transformed into expression host *E. coli* BL21(DE3). Eight to twenty colonies expressing FP genes were selected and sequenced (GeneWiz). Sequence data were analysed using CLC Bio software version 4.1.2. The average PCR FP variant contained 1–4 base substitutions per round of random mutagenesis given the conditions above (conditions optimized for this mutation load). One mutant was retained after each round of mutagenesis and used for subsequent rounds of random mutagenesis. Mutants were selected to balance the frequency of synonymous and nonsynonymous substitutions along branches of the experimental gene phylogeny. In some instances, two mutants were retained after a round of mutagenesis to bifurcate the phylogeny. The experimental phylogeny was initiated by replacing seven amino acid positions known to affect emission phenotype^32^ (Supplementary Fig. 1), all other mutations were random. All leaf and internal node amino acid sequences are proved (Supplementary Note 1).

### Ancestral sequence reconstruction

The 19 ‘modern' sequences at the tips (leaves) of the FP phylogeny were used to computationally reconstruct infer ancestral sequences at all internal nodes of the tree using the evolved (known) topology. Marginally reconstructed ancestral sequences were inferred using Bayesian approaches that incorporated the WAG amino acid replacement matrix (PAML, FastML and PhyloBayes [CAT]), with or without rate variation as modelled by a discrete gamma distribution (four rate categories), and ancestral sequences were also inferred with the MP criterion (as implemented in PAML). DNA and codon-based analyses were performed only in PAML using HKY85+GC and M0(F3x4), respectively. ProtTest v3.2 was used to analyse the various models according to the AIC criterion (AIC weight was 100% for WAG_Г)^33^.

### Protein expression and purification

FP variants were transformed into BL21(DE3) bacterial cells. Transformed cells grew overnight at 37 °C on LB-agar supplemented with 50 μg ml^−1^ carbenicillin. A single colony was inoculated and grown overnight in 5 ml LB/carbenicillin. The next day, the culture was used to inoculate LB/carbenicillin using a 3:100 ratio. Cells were induced at OD_600_ of 0.55–0.9 with isopropyl β-D-1-thiogalactopyranoside (IPTG) to a final concentration of 100 μM. Cultures continued to grow for 4–6 h at 37 °C and were then harvested by centrifugation and stored at −80 °C. Cell pellets were lysed with BugBuster (Novagen) and purified by Ni-NTA agarose (Qiagen) in elution buffer containing 500 mM imidazole per manufacturer's protocol. Imidazole was removed from the protein samples via buffer exchange using 20-kDa concentrators (Pierce) or dialysis into 50 mM Tris buffer pH 7.5. Purity of protein sample was assessed by SDS–PAGE.

### Protein characterization and spectroscopic studies

Absorption spectra of purified protein were recorded on a Varian Cary 50 ultraviolet–visible spectrophotometer (Supplementary Fig. 5). Excitation and emission spectra were recorded on a Varian Cary Eclipse fluorescence spectrophotometer. All measurements were performed in quartz cuvettes at ambient temperature and purified protein sample was diluted in 5 mM Tris/HCl (pH 7.5). Quantum yield experiments were performed as described^34^,^35^ and variant proteins were compared to equally absorbing solutions of mRFP1 for red emitting variants, TagGFP2 for yellow and orange emitting variants, or TagBFP for green and blue emitting variants. Quantum yield values were computed using a|e UV–vis-IR Spectral software. Extinction coefficients were determined as described^10^. Molar extinction coefficient values were calculated using the maximum absorbance at wavelength of maximum excitation.

The relative errors between the inferences across all nodes for each procedure versus the true ancestors were determined for extinction coefficient, quantum yield and brightness. Bootstrap analysis were performed; the actual sum of the differences in relative error for each node between two methods was first determined. Then, a distribution of sums of differences in relative error for each node between any two procedures was made for 100 bootstrap replicate data sets generated by assigning error values from the appropriate nodes of the original data set with replacement. The percentile of the actual sum of differences in relative error in the distribution was determined and reported.

### Data availability

All relevant data are available from the authors by request.

## Additional information

**How to cite this article**: Randall, R. N. *et al.* An experimental phylogeny to benchmark ancestral sequence reconstruction. *Nat. Commun.* 7:12847 doi: 10.1038/ncomms12847 (2016).

## Supplementary Material

Supplementary Information Supplementary Figures 1 - 5, Supplementary Tables 1 - 3 and Supplementary Note 1
