# Supplementary material for: An experimental phylogeny to benchmark ancestral sequence reconstruction
Source: Nat Commun. 2016 Sep 15;7:12847. doi: 10.1038/ncomms12847 (PMC5027606; doi:10.1038/ncomms12847)
Supplement: Supplementary Information — Supplementary Figures 1 - 5, Supplementary Tables 1 - 3 and Supplementary Note 1 [file ncomms12847-s1.pdf]

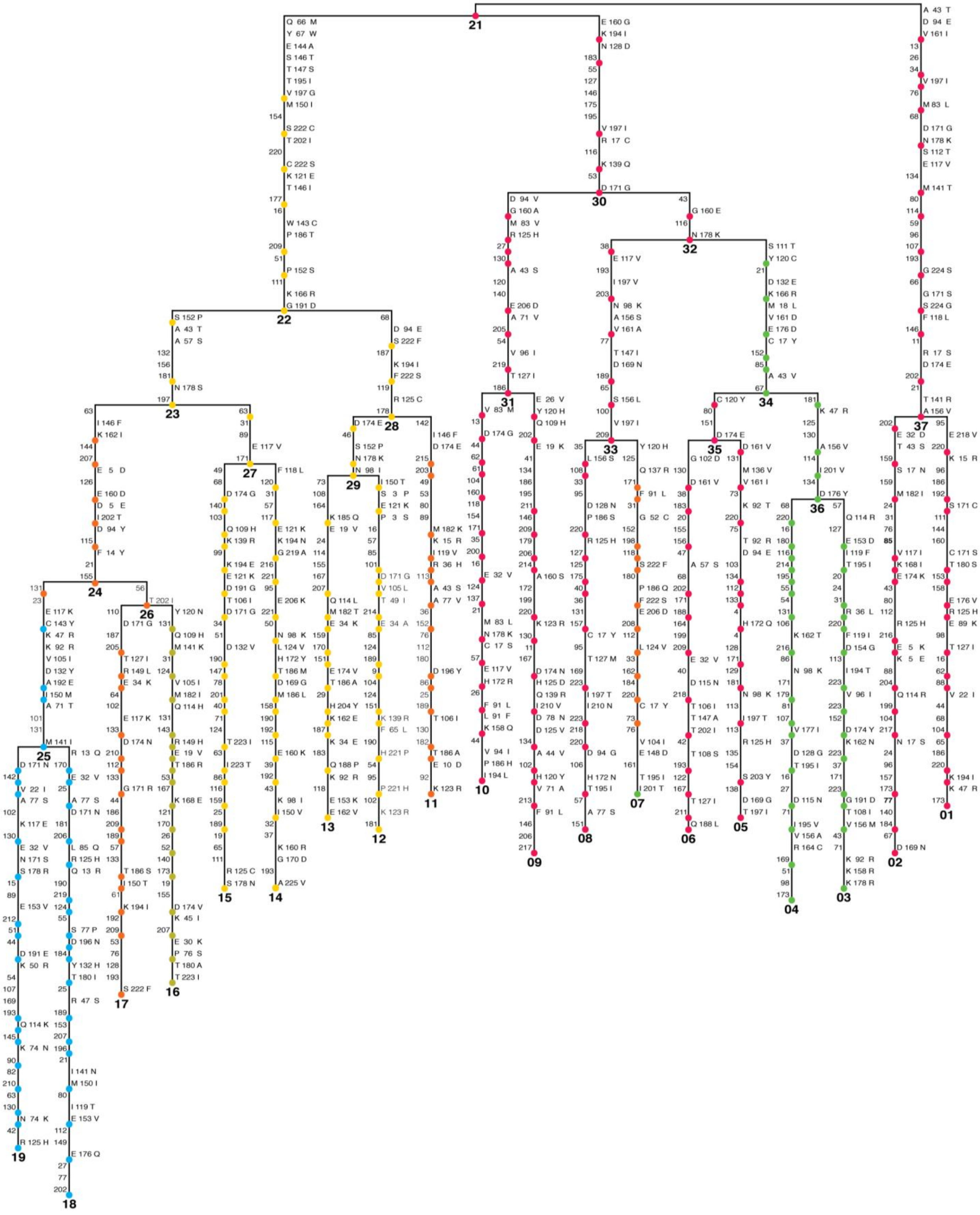
21
22
23
24
25
26
27
28
29
30
31
32
33
34
35
36
37
01
02
03
04
05
06
07
08
09
10
11
12
13
14
15
16
17
18
19

Supplementary Figure 1. Distribution of mutations in the experimental phylogeny. Leaf and node numbers are provided at each tip and bifurcation point on the tree, respectively. Nonsynonymous mutations are listed to the right of each branch and synonymous mutations to the left. Nonsynonymous mutations also list the amino acid replacement. Each filled circle represents one round of random-mutagenesis PCR and the color of the circle represents the color-class phenotype of the FP protein at that location in the tree.

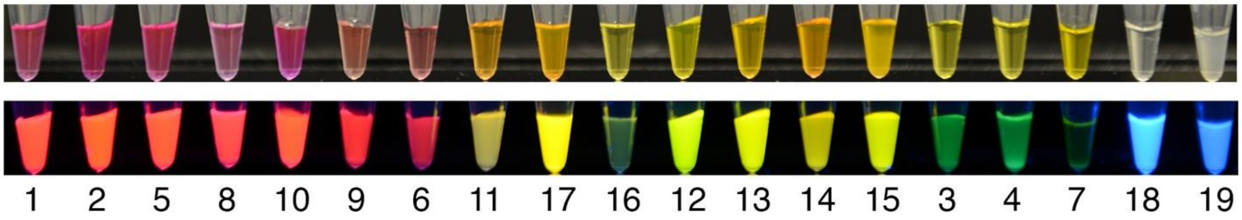

Supplementary Figure 2. Diversity of phenotypes displayed by the leaf ('modern') sequences of the phylogeny. Purified proteins from sequences 1-19 are arranged by color-class phenotype and are shown in visible light (top image) and 365 nm UV light (bottom image).

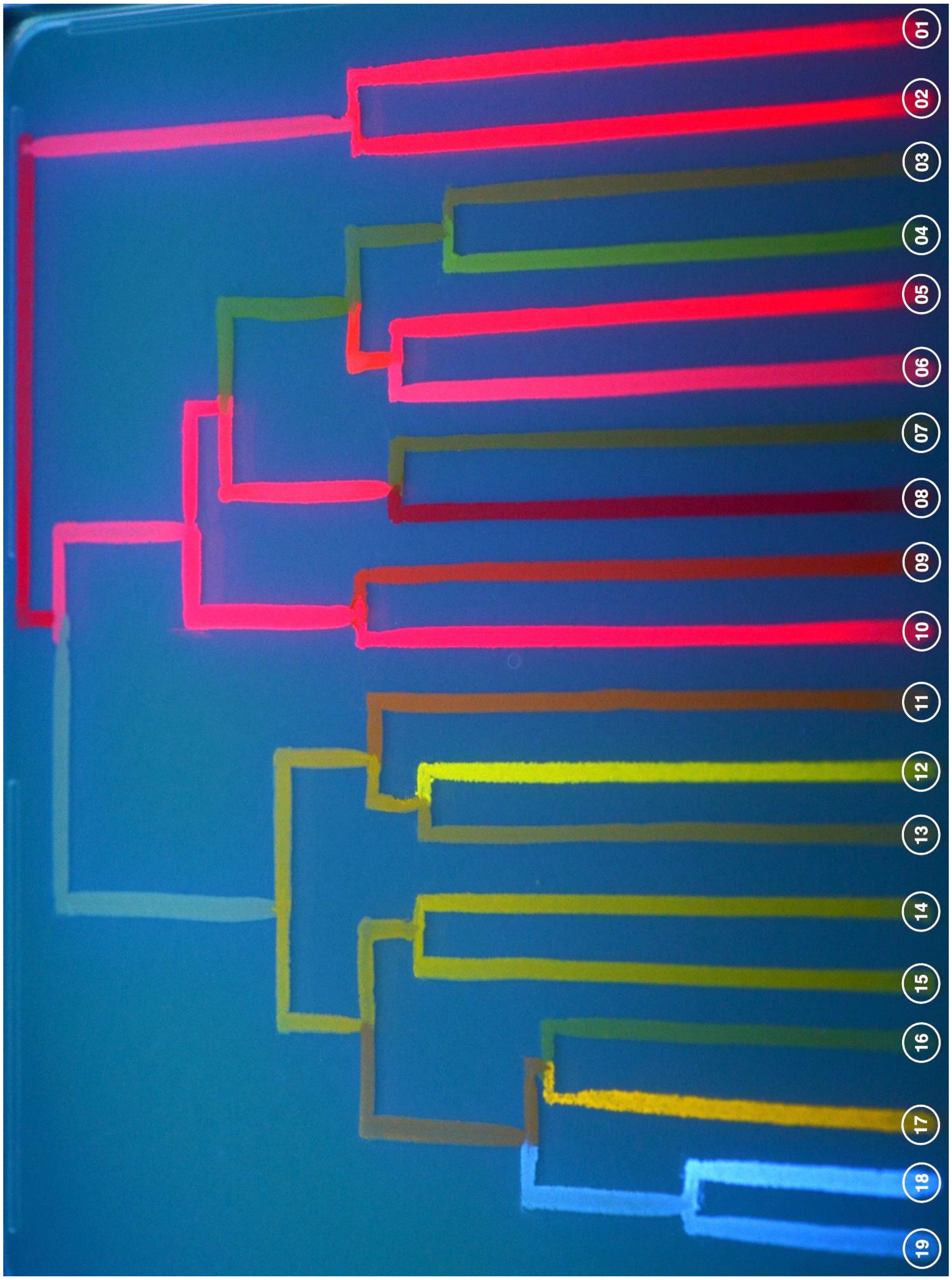

Supplementary Figure 3. Evolution of color emission in the experimental phylogeny. Cladogram drawn with bacteria growing on an agar plate expressing either a node or leaf fluorescent protein and visualized under 365 nm ultraviolet light. Topology is a representation of the true topology shown in the phylogram (Supplementary Fig. 1).

a

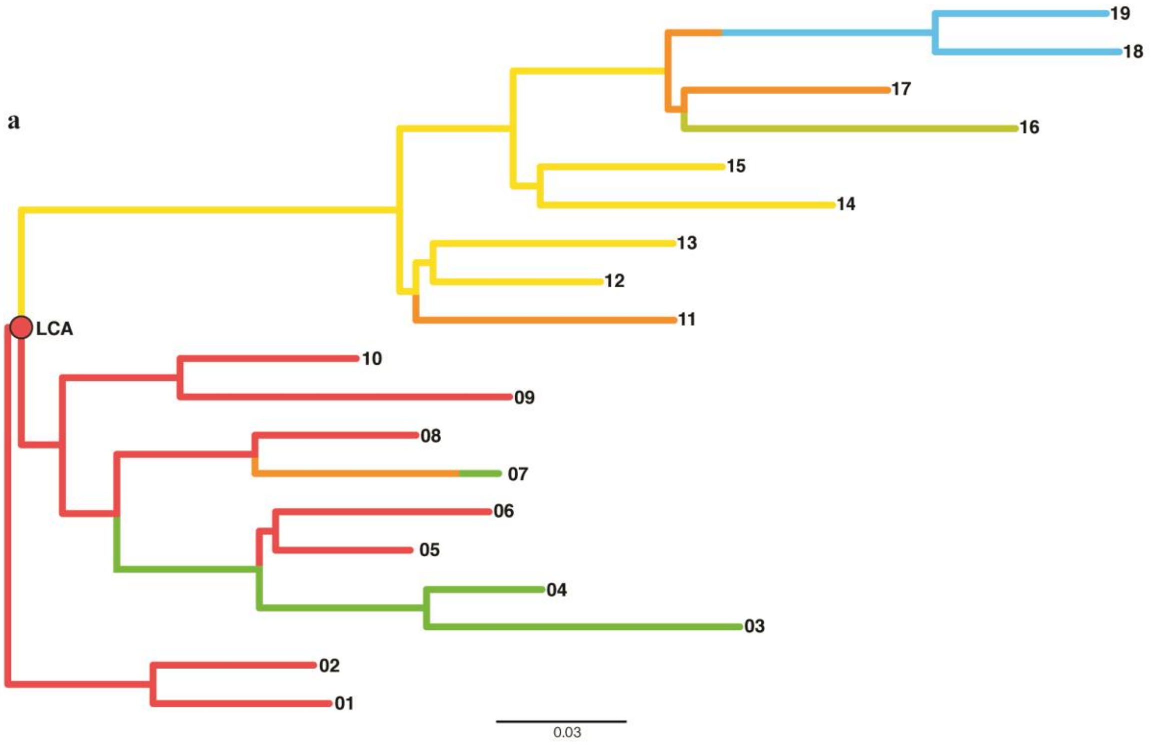

19
18
17
16
15
14
13
12
11
LCA
10
09
08
07
06
05
04
03
02
01
0.03

b

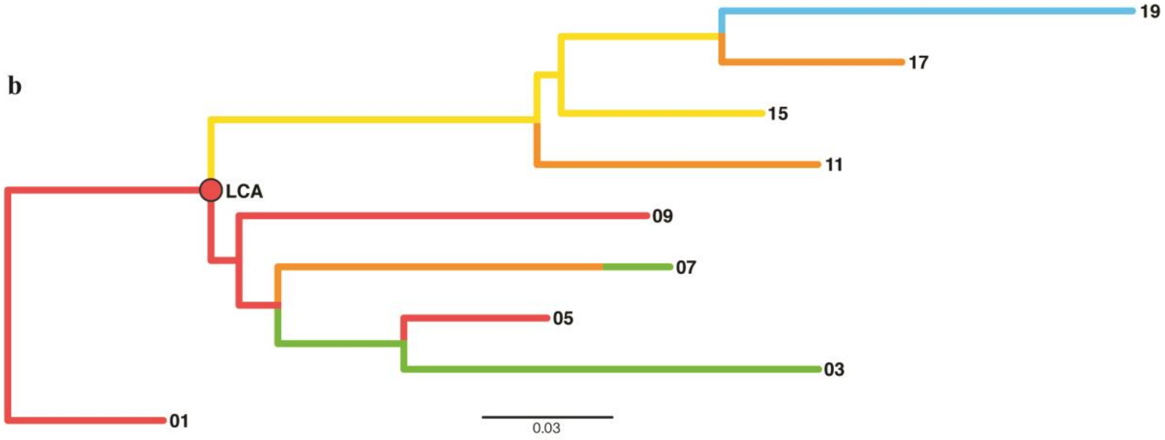

19
17
15
11
LCA
09
07
05
03
01
0.03

c

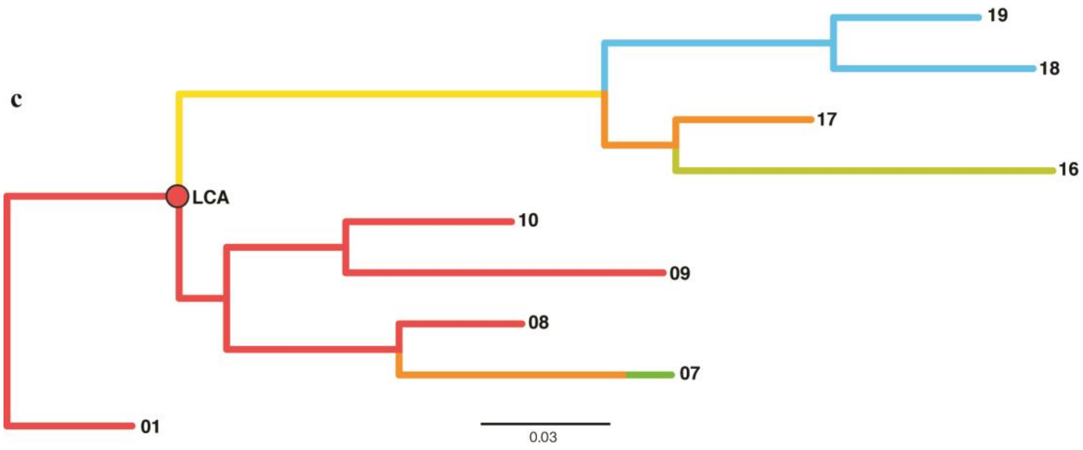

19
18
17
16
LCA
10
09
08
07
01
0.03

d

```
TrueLCA         MASSEDVIKEFMRFKVRMEGSVNGHEFEIEGEGEGRPYEGTQAAKLKVTKGGPLPFAWDI
PAML all 19     MASSEDVIKEFMRFKVSMEGSVNGHEFEIEGEGEGRPYEGTQSAKLKVTKGGPLPFAWDI
PAML Sub1       MASSEDVIKEFMRFKVSMEGSVNGHEFEIEGEGEGRPYEGTQTAKLKVTKGGPLPFAWDI
PAML Sub2       MASSEDVIKEFMRFKVSMEGSVNGHEFEIEGEGEGRPYEGTQTAKLKVTKGGPLPFAWDI
                **************** *************************:*****************

TrueLCA         LSPQFQYGSKAYVKHPADIPDYMKLSFPEGFKWDRVMNFEDGGVVTVTQDSSLQDGEFIY
PAML all 19     LSPQFQYGSKAYVKHPADIPDYMKLSFPEGFKWERVMNFEDGGVVTVTQDSSLQDGVFIY
PAML Sub1       LSPQFQYGSKAYVKHPADIPDYMKLSFPEGFKWERVMNFEDGGVVTVTQDSSLQDGEFIY
PAML Sub2       LSPQFQYGSKAYVKHPADIPDYMKLSFPEGFKWERVMNFEDGGVVTVTQDSSLQDGVFIY
                *********************************:********************** ***

TrueLCA         KVKLRGTNFPSDGPVMQKKTMGWEASTERMYPEDGALKGEVKMRLKLKDGDHYDAEVNTT
PAML all 19     KVKLHGTNFPSDGPVMQKKTMGWEASTERMYPEDGALKGEVKMRLKLKDGGHYEAEVKTT
PAML Sub1       KVKLHGTNFPSDGPVMQKKTMGWEASTERMYPEDGALKGEVKMRLKLKDGGHYEAEVKTT
PAML Sub2       KVKLHGINFPSDGPVMQKKTMGWEASTERMYPEDGALKGEVKMRLKLKDGSHYDAEVKTT
                ****.* *******************************************.**:***:**

TrueLCA         YMAKKPVQLPGAYKTDVKLDITSHNEDYTIVEQYERAEGRHSTGA
PAML all 19     YMAKKPVQLPGAYITDIKLDITSHNEDYTIVEQYERAEGRHSTGA
PAML Sub1       YMAKKPVQLPGAYITDIKLDITSHNEDYTIVEQYERAEGRHSTGA
PAML Sub2       YMAKKPVQLPGAYITDIKLDITSHNEDYTIVEQYERAEGRHSTGA
                ************* **:****************************
```

Supplementary Figure 4. The effects of taxon sampling on the inference of the last common ancestral sequence. (**a**) Full phylogeny from Fig. 1 in the main article. (**b**) Subsample of sequences across the entire spectrum of the full phylogeny used to infer the last common ancestor (LCA). (**c**) Subsample of sequences composed of clusters from the full phylogeny used to infer the LCA. Scale bars represents amino acid replacements per site per unit evolutionary time. Color corresponds to Fig.1 from the main article. (**d**) Multiple sequence alignment of LCAs from the different analyses; true ancestor, PAML inferred using all of the 19 leaf sequences, PAML using sequences from **b** (Subsample1), PAML using sequences from **c** (Subsample2). Inferred residues that differ from the true ancestral residues are highlighted in magenta. The sites of the highlighted positions are 17, 43, 94, 117, 125, 127, 171, 174, 178, 194 and 197.

a

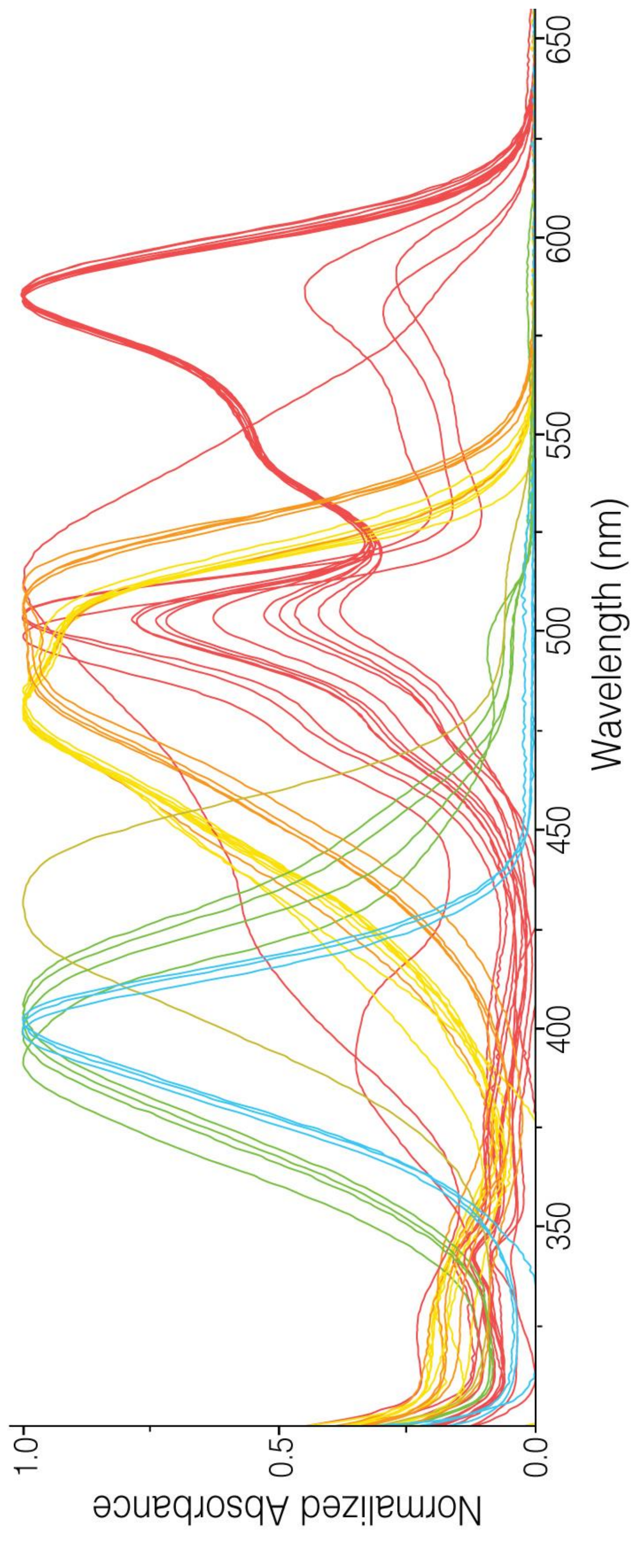

Normalized Absorbance
1.0
0.5
0.0
Wavelength (nm)
350
400
450
500
550
600
650

b

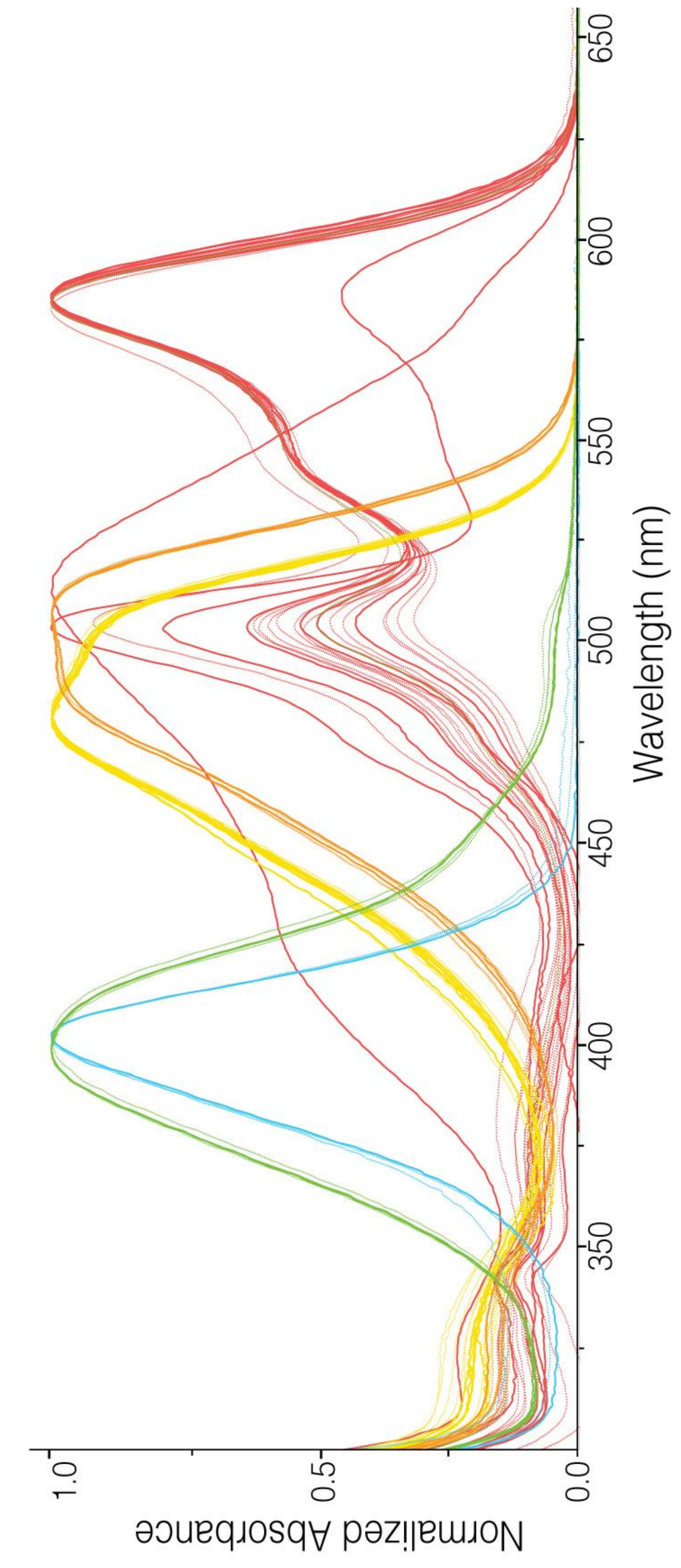
Normalized Absorbance
1.0
0.5
0.0
350
400
450
500
550
600
650
Wavelength (nm)

Supplementary Figure 5. Absorbance spectra for all 70 proteins characterized in the study. (**a**) Absorbance spectra for true ancestors and leaves of phylogeny. Color of spectral line represents color-class of encoded protein. (**b**) Absorbance spectra for true ancestors (solid lines) and incorrectly inferred ancestors (dotted lines).

Supplementary Table 1. Summary statistics for the experimental phylogeny. (**a**) Total mutations and types of mutations accumulated in the phylogeny. (**b**) A list of specific nucleotide substitution types and occurrences in the phylogeny. Abbreviations ts and tv represent transitions and transversions, repectively. (**c**) Types of nucleotide substitutions resulting in either a synonymous (syn) or nonsynonymous (nonsyn) mutation along each individual branch of the phylogeny. Predicted ratio of nonsynonymous to synonymous mutations does not match the observed ratio because of reversion mutations.

a

**Experimental Phylogeny Mutation Summary**	
Total variants in phylogeny	349
Nodes	17
Leaves	19
Total Mutations	833
Transitions (ts)	535
Transversions (tv)	298
Total synonymous (syn) mutations	461
Synonymous reversions	14
Observed synonymous mutations	447
Total nonsynonymous (nonsyn) mutations	372
Nonsynonymous reversions	19
Observed nonsynonymous mutations	353

b

**Nucleotide Substitution**			
Substitution	Type	Occurrence	Percentage (%)
A --> T	tv	74	8.88
A --> C	tv	16	1.92
A --> G	ts	117	14.05
T --> A	tv	72	8.64
T --> C	ts	106	12.73
T --> G	tv	19	2.28
C --> T	ts	167	20.05
C --> G	tv	17	2.04
C --> A	tv	33	3.96
G --> C	tv	21	2.52
G --> T	tv	46	5.52
G --> A	ts	145	17.41

c

Branch number	Syn mutations	Syn reversions	Observed syn mutations	Nonsyn mutations	Nonsyn reversions	Observed nonsyn mutations	Observed nonsyn/syn	Predicted nonsyn/syn
1	21	0	21	12	1	11	0.5714	0.5238
2	24	1	23	13	2	11	0.5417	0.4783
3	16	2	14	17	1	16	1.0625	1.1429
4	25	0	25	9	0	9	0.3600	0.3600
5	19	1	18	13	1	12	0.6842	0.6667
6	21	0	21	11	0	11	0.5238	0.5238
7	16	0	16	14	1	13	0.8750	0.8125
8	21	1	20	12	0	12	0.5714	0.6000
9	23	2	21	16	1	15	0.6957	0.7143
10	18	1	17	14	1	13	0.7778	0.7647
11	19	0	19	13	0	13	0.6842	0.6842
12	17	0	17	13	2	11	0.7647	0.6471
13	15	0	15	14	1	13	0.9333	0.8667
14	19	2	17	17	0	17	0.8947	1.0000
15	23	0	23	13	1	12	0.5652	0.5217
16	16	1	15	16	0	16	1.0000	1.0667
17	22	1	21	11	0	11	0.5000	0.5238
18	21	1	20	17	1	16	0.8095	0.8000
19	19	1	18	14	1	13	0.7368	0.7222
22	7	0	7	20	1	19	2.8571	2.7143
23	4	0	4	4	0	4	1.0000	1.0000
24	7	0	7	8	1	7	1.1429	1.0000
25	4	0	4	10	0	10	2.5000	2.5000
26	1	0	1	1	0	1	1.0000	1.0000
27	4	0	4	1	0	1	0.2500	0.2500
28	4	0	4	5	1	4	1.2500	1.0000
29	1	0	1	4	0	4	4.0000	4.0000
30	8	0	8	7	0	7	0.8750	0.8750
31	8	0	8	9	0	9	1.1250	1.1250
32	2	0	2	2	0	2	1.0000	1.0000
33	8	0	8	9	1	8	1.1250	1.0000
34	4	0	4	9	0	9	2.2500	2.2500
35	2	0	2	2	0	2	1.0000	1.0000
36	5	0	5	4	0	4	0.8000	0.8000
37	17	0	17	18	1	17	1.0588	1.0000
						Average	1.0510	1.0267

Supplementary Table 2. Percentage of sites correctly inferred for the five tested ASR procedures. Procedures follow the color-code scheme (Fig. 2 in main article).

Method	Correctly Inferred Sites	Incorrectly Inferred Sites	Total Sites
PAML_Γ	98.14%	71	3825
FASTML_Γ	98.17%	70	3825
PAML	98.12%	72	3825
PHYLO_Γ	97.88%	81	3825
MP	98.07%	74	3825

Supplementary Table 3. (**a**) Phenotypes of true ancestors and incorrectly inferred ancestors. Extinction coefficient (ε), quantum yield (Φ), brightness (ε x Φ), excitation maxima (λ_{ex}), and emission maxima (λ_{em}). The percent error equals zero when the inferred sequence is identical to the ancestral sequence. (**b**) Phenotypes for all 19 leaf proteins. (**c**) Summary of phenotypic error for each ASR procedure. Note, nodes 34 and 35 are not included because they outweigh all other error (but their errors are the same for the five procedures so the overall effect is negligible. Procedures follow the color-code scheme (Supplementary Table 2a, c, Fig. 2).

a

Node	Σ ($M^{-1}cm^{-1}$)	Σ Error	√	√ Error	λ_{ex} (nm)	λ_{ex} Error	λ_{em} (nm)	λ_{em} Error	Brightness	Brightness Error
21	19,003	-	0.1213	-	586	-	608	-	2,306	-
PAML_Γ	36,317	91.11%	0.2567	111.53%	585	0.17%	604	0.66%	9,322	304.25%
FASTML_Γ	36,317	91.11%	0.2567	111.53%	585	0.17%	604	0.66%	9,322	304.25%
PAML	36,317	91.11%	0.2567	111.53%	585	0.17%	604	0.66%	9,322	304.25%
PHYLO_Γ	36,317	91.11%	0.2567	111.53%	585	0.17%	604	0.66%	9,322	304.25%
MP	46,312	143.71%	0.2532	108.62%	585	0.17%	606	0.33%	11,724	408.43%
22	7,307	-	0.0710	-	486	-	549	-	519	-
PAML_Γ	18,524	153.51%	0.0659	7.22%	486	0.00%	546	0.55%	1,221	135.21%
FASTML_Γ	18,524	153.51%	0.0659	7.22%	486	0.00%	546	0.55%	1,221	135.21%
PAML	19,352	164.84%	0.0768	8.09%	486	0.00%	546	0.55%	1,486	186.26%
PHYLO_Γ	19,352	164.84%	0.0768	8.09%	486	0.00%	546	0.55%	1,486	186.26%
MP	18,276	150.12%	0.0656	7.60%	486	0.00%	546	0.55%	1,199	131.11%
23	16,329	-	0.0803	-	486	-	546	-	1,312	-
PAML_Γ	16,443	0.70%	0.0770	4.18%	486	0.00%	546	0.00%	1,266	3.51%
FASTML_Γ	16,443	0.70%	0.0770	4.18%	486	0.00%	546	0.00%	1,266	3.51%
PAML	20,125	23.25%	0.0809	0.74%	486	0.00%	546	0.00%	1,628	24.16%
PHYLO_Γ	20,125	23.25%	0.0809	0.74%	486	0.00%	546	0.00%	1,628	24.16%
MP	18,353	12.40%	0.0773	3.71%	486	0.00%	546	0.00%	1,419	8.23%
24	24,473	-	0.0713	-	512	-	560	-	1,745	-
PAML_Γ	26,404	7.89%	0.0831	16.49%	511	0.20%	560	0.00%	2,193	25.69%
FASTML_Γ	26,404	7.89%	0.0831	16.49%	511	0.20%	560	0.00%	2,193	25.69%
PAML	26,404	7.89%	0.0831	16.49%	511	0.20%	560	0.00%	2,193	25.69%
PHYLO_Γ	25,695	4.99%	0.0805	12.87%	512	0.00%	561	0.18%	2,068	18.50%
MP	25,486	4.14%	0.0769	7.93%	512	0.00%	560	0.00%	1,961	12.40%
25	17,857	-	0.0868	-	373	-	452	-	1,550	-
PAML_Γ	11,018	38.30%	0.1036	19.39%	399	6.97%	451	0.22%	1,142	26.34%
FASTML_Γ	11,018	38.30%	0.1036	19.39%	399	6.97%	451	0.22%	1,142	26.34%
PAML	24,857	39.20%	0.0989	13.90%	400	7.24%	451	0.22%	2,458	58.55%
PHYLO_Γ	24,857	39.20%	0.0989	13.90%	400	7.24%	451	0.22%	2,458	58.55%
MP	11,018	38.30%	0.1036	19.39%	399	6.97%	451	0.22%	1,142	26.34%
26	25,592	-	0.0720	-	512	-	560	-	1,842	-
PAML_Γ	25,592	0.00%	0.0720	0.00%	512	0.00%	560	0.00%	1,842	0.00%
FASTML_Γ	25,592	0.00%	0.0720	0.00%	512	0.00%	560	0.00%	1,842	0.00%
PAML	22,798	10.92%	0.0756	5.08%	511	0.20%	560	0.00%	1,725	6.39%
PHYLO_Γ	26,469	3.43%	0.0708	1.64%	512	0.00%	560	0.00%	1,874	1.74%
MP	25,486	0.41%	0.0769	6.89%	512	0.00%	560	0.00%	1,961	6.45%
27	18,419	-	0.0758	-	486	-	546	-	1,396	-
PAML_Γ	20,125	9.26%	0.0809	6.78%	486	0.00%	546	0.00%	1,628	16.67%
FASTML_Γ	20,125	9.26%	0.0809	6.78%	486	0.00%	546	0.00%	1,628	16.67%
PAML	20,125	9.26%	0.0809	6.78%	486	0.00%	546	0.00%	1,628	16.67%
PHYLO_Γ	20,125	9.26%	0.0809	6.78%	486	0.00%	546	0.00%	1,628	16.67%
MP	16,939	8.04%	0.0744	1.75%	486	0.00%	546	0.00%	1,261	9.64%
28	17,288	-	0.0769	-	486	-	545	-	1,329	-
PAML_Γ	18,524	7.15%	0.0659	14.28%	486	0.00%	546	0.18%	1,221	8.15%
FASTML_Γ	18,524	7.15%	0.0659	14.28%	486	0.00%	546	0.18%	1,221	8.15%
PAML	18,524	7.15%	0.0659	14.28%	486	0.00%	546	0.18%	1,221	8.15%
PHYLO_Γ	15,953	7.72%	0.0753	2.01%	486	0.00%	546	0.18%	1,202	9.58%

MP	18,276	5.71%	0.0656	14.63%	486	0.00%	546	0.18%	1,199	9.75%
29	18,034	-	0.0803	-	487	-	547	-	1,448	-
PAML_Γ	17,102	5.17%	0.0775	3.54%	487	0.00%	547	0.00%	1,325	8.53%
FASTML_Γ	17,102	5.17%	0.0775	3.54%	487	0.00%	547	0.00%	1,325	8.53%
PAML	17,102	5.17%	0.0775	3.54%	487	0.00%	547	0.00%	1,325	8.53%
PHYLO_Γ	19,237	6.67%	0.0815	1.43%	486	0.21%	547	0.00%	1,567	8.20%
MP	18,034	0.00%	0.0803	0.00%	487	0.00%	547	0.00%	1,448	0.00%
30	26,360	-	0.2398	-	586	-	606	-	6,322	-
PAML_Γ	32,151	21.97%	0.2486	3.67%	586	0.00%	604	0.33%	7,993	26.44%
FASTML_Γ	32,151	21.97%	0.2486	3.67%	586	0.00%	604	0.33%	7,993	26.44%
PAML	32,151	21.97%	0.2486	3.67%	586	0.00%	604	0.33%	7,993	26.44%
PHYLO_Γ	37,090	40.71%	0.2489	3.77%	585	0.17%	605	0.17%	9,231	46.01%
MP	39,835	51.12%	0.2547	6.19%	586	0.00%	605	0.17%	10,145	60.48%
31	36,941	-	0.2528	-	584	-	602	-	9,339	-
PAML_Γ	33,535	9.22%	0.2514	0.54%	584	0.00%	604	0.33%	8,431	9.71%
FASTML_Γ	33,535	9.22%	0.2514	0.54%	584	0.00%	604	0.33%	8,431	9.71%
PAML	45,841	24.09%	0.2437	3.60%	585	0.17%	605	0.50%	11,171	19.62%
PHYLO_Γ	45,841	24.09%	0.2437	3.60%	585	0.17%	605	0.50%	11,171	19.62%
MP	49,196	33.17%	0.2527	0.03%	585	0.17%	605	0.50%	12,433	33.14%
32	38,038	-	0.2527	-	586	-	608	-	9,614	-
PAML_Γ	31,981	15.92%	0.2483	1.76%	585	0.17%	604	0.66%	7,941	17.40%
FASTML_Γ	31,981	15.92%	0.2483	1.76%	585	0.17%	604	0.66%	7,941	17.40%
PAML	31,981	15.92%	0.2483	1.76%	585	0.17%	604	0.66%	7,941	17.40%
PHYLO_Γ	28,842	24.18%	0.2616	3.52%	585	0.17%	605	0.49%	7,546	21.51%
MP	31,981	15.92%	0.2483	1.76%	585	0.17%	604	0.66%	7,941	17.40%
33	30,069	-	0.2572	-	584	-	604	-	7,733	-
PAML_Γ	30,873	2.67%	0.2178	15.31%	583	0.17%	604	0.00%	6,724	13.05%
FASTML_Γ	30,873	2.67%	0.2178	15.31%	583	0.17%	604	0.00%	6,724	13.05%
PAML	30,873	2.67%	0.2178	15.31%	583	0.17%	604	0.00%	6,724	13.05%
PHYLO_Γ	30,873	2.67%	0.2178	15.31%	583	0.17%	604	0.00%	6,724	13.05%
MP	30,873	2.67%	0.2178	15.31%	583	0.17%	604	0.00%	6,724	13.05%
34	26,491	-	0.0019	-	402	-	512	-	51	-
PAML_Γ	30,213	14.05%	0.2326	11927.88%	584	45.27%	604	17.97%	7,028	13617.80%
FASTML_Γ	30,213	14.05%	0.2326	11927.88%	584	45.27%	604	17.97%	7,028	13617.80%
PAML	30,213	14.05%	0.2326	11927.88%	584	45.27%	604	17.97%	7,028	13617.80%
PHYLO_Γ	30,213	14.05%	0.2326	11927.88%	584	45.27%	604	17.97%	7,028	13617.80%
MP	30,213	14.05%	0.2326	11927.88%	584	45.27%	604	17.97%	7,028	13617.80%
35	8,629	-	0.0069	-	585	-	605	-	60	-
PAML_Γ	30,213	250.13%	0.2326	3270.90%	584	0.17%	604	0.17%	7,028	11702.63%
FASTML_Γ	30,213	250.13%	0.2326	3270.90%	584	0.17%	604	0.17%	7,028	11702.63%
PAML	30,213	250.13%	0.2326	3270.90%	584	0.17%	604	0.17%	7,028	11702.63%
PHYLO_Γ	31,380	263.66%	0.2481	3496.06%	583	0.34%	603	0.33%	7,787	12977.33%
MP	30,213	250.13%	0.2326	3270.90%	584	0.17%	604	0.17%	7,028	11702.63%
36	24,031	-	0.0010	-	406	-	511	-	23	-
PAML_Γ	25,263	5.13%	0.0010	1.54%	400	1.48%	511	0.00%	25	6.75%
FASTML_Γ	27,819	15.76%	0.0011	12.79%	400	1.48%	511	0.00%	30	30.57%
PAML	27,819	15.76%	0.0011	12.79%	400	1.48%	511	0.00%	30	30.57%
PHYLO_Γ	27,819	15.76%	0.0011	12.79%	400	1.48%	511	0.00%	30	30.57%
MP	27,266	13.46%	0.0021	119.23%	400	1.48%	511	0.00%	57	148.74%
37	39,333	-	0.2905	-	584	-	604	-	11,425	-
PAML_Γ	49,835	26.70%	0.2904	0.04%	584	0.00%	603	0.17%	14,470	26.65%
FASTML_Γ	49,835	26.70%	0.2904	0.04%	584	0.00%	603	0.17%	14,470	26.65%
PAML	49,835	26.70%	0.2904	0.04%	584	0.00%	603	0.17%	14,470	26.65%
PHYLO_Γ	49,835	26.70%	0.2904	0.04%	584	0.00%	603	0.17%	14,470	26.65%
MP	49,835	26.70%	0.2904	0.04%	584	0.00%	603	0.17%	14,470	26.65%

b

Leaf	ε(M^{-1}cm^{-1})	Φ	λ_{ex} (nm)	λ_{em} (nm)	Brightness
1	43,663	0.2819	585	604	12,309
2	50,293	0.2837	584	602	14,270
3	26,750	0.0036	398	511	96
4	26,759	0.0052	425	513	140
5	61,727	0.2717	584	605	16,773
6	38,793	0.2541	584	605	9,858
7	30,084	0.0008	497	511	24
8	19,474	0.0747	580	604	1,455
9	8,572	0.2321	589	609	1,990
10	46,478	0.2412	584	603	11,208
11	27,193	0.0544	509	557	1,480
12	17,339	0.1335	486	532	2,314
13	11,352	0.0716	486	548	813
14	17,366	0.0951	486	543	1,651
15	13,477	0.0939	486	533	1,265
16	23,978	0.0061	510	565	145
17	25,406	0.1648	512	556	4,186
18	29,356	0.1348	398	451	3,956
19	10,572	0.1038	399	450	1,097

c

**Method**	**ε Average Error**	**Φ Average Error**	**Brightness Average Error**
PAML_Γ	26.31%	13.75%	41.89%
FASTML_Γ	27.02%	14.50%	43.48%
PAML	31.06%	14.51%	51.49%
PHYLO_Γ	32.31%	13.20%	52.36%
MP	33.73%	20.87%	60.79%

Nodes 34 and 35 not included due to being outliers

Supplementary Note 1

Amino acid sequences for the 37 proteins at the leaves and nodes in the experimental phylogeny.

>01
MASSEDVIKEFMRFRVSMEGSINGHEFEIEGEGEGRPYEGTQTAKLRVTKGGPLPFAWDILSPQF
QYGSKAYVKHPADIPDYLKLSFPKGFKWERVMNFEDGGVVTVTQDSTLQDGVLIYKVKLHGINFP
SDGPVMQKKTRGWEASTERMYPEDGVLKGEIKMRLKLKDGSHYEAVVKTSYMAKKPVQLPGAYIT
DIKLDITSHNEDYTIVEQYERAVGRHSTGA

>02
MASSEDVIKEFMRFKVSMEGSVNGHEFEIEGDGEGRPYEGTQSAKLKVTKGGPLPFAWDILSPQF
QYGSKAYVKHPADIPDYLKLSFPEGFKWERVMNFEDGGVVTVTQDSTLRDGILIYKVKLHGTNFP
SDGPVMQKKTRGWEASTERMYPEDGVLKGEIKMRLKLINGSHYKAEVKTTYIAKKPVQLPGAYKT
DIKLDITSHNEDYTIVEQYERAEGRHSTGA

>03
MASSEDVIKEFMRFKVYLEGSVNGHEFEIEGEGEGLPYEGTQVAKLRVTKGGPLPFAWDILSPQF
QYGSKAYVKHPADIPDYMKLSFPEGFRWDRIMNFEDGGVVTVIQDTSLRDGEFICKVKLRGTDFP
SEGPVMQKQTMGWEASTERMYPDGGMLRGEDNMRLRLKDGGHYYAYVRTTYMAKKPVQLPDAYTI
DIKLDVTSHNEDYTIVEQYERAEGRHSTGA

>04
MASSEDVIKEFMRFKVYLEGSVNGHEFEIEGEGEGRPYEGTQVAKLRVTKGGPLPFAWDILSPQF
QYGSKAYVKHPADIPDYMKLSFPEGFKWDRVMKFEDGGVVTVTQDTSLQNGEFICKVKLRGTGFP
SEGPVMQKQTMGWEASTERMYPEDGALKGEDTMCLRLKDGGHYDAYIKTTYMAKKPVQLPGAYIV
DIKLDVTSHNEDYTIVEQYERAEGRHSTGA

>05
MASSEDVIKEFMRFKVYLEGSVNGHEFEIEGEGEGRPYEGTQVAKLKVTKGGPLPFAWDILSPQF
QYGSKAYVKHPADIPDYMKLSFPEGFRWERVMKFEDGGVVTVTQDTSLQDGEFIYKVKLHGTDFP
SEGPVVQKQTMGWEASTERMYPEDGALKGEIKMRLRLKGGGQYEADVKTTYMAKKPVQLPGAYIT
DIKLDITYHNEDYTIVEQYERAEGRHSTGA

>06
MASSEDVIKEFMRFKVYLEGSVNGHEFEIEGVGEGRPYEGTQVAKLKVTKGGPLPFSWDILSPQF
QYGSKAYVKHPADIPDYMKLSFPEGFKWDRVMNFEDDGVVIVSQDTSLQNGEFIYKVKLRGIDFP
SEGPVMQKQTMGWEASAERMYPEDGALKGEVKMRLRLKDGGHYEADVKTTYMAKKPVLLPGAYIT
DIKLDIISHNEDYTIVEQYERAEGRHSTGA

>07
MASSEDVIKEFMRFKVYMEGSVNGHEFEIEGEGEGRPYEGTQAAKLKVTKGCPLPFAWDILSPQF
QYGSKAYVKHPADIPDYMKLSFPEGLKWDRVMKFEDGGIVTVTQDSSLQDGVFIHKVKVRGTDFP
SDGPVMRKQTMGWEASIDRMYPEDGLLKGEAKMRLKLKNGGHYDAEVKTTYMAKKQVQLPGAYII
DIKLDTTSHNDDYTIVEQYERAEGRHSTGA

>08
MASSEDVIKEFMRFKVYMEGSVNGHEFEIEGEGEGRPYEGTQAAKLKVTKGGPLPFAWDILSPQF
QYGSKAYVKHPSDIPDYMKLSFPEGFKWGRVMKFEDGGVVTVTQDSSLQDGVFIYKVKLHGMNFP
SDGPVMQKQTMGWEASIERMYPEDGSLKGEAKMRLKLKNGGNYDAEVKTTYMAKKSVQLPGAYII
DTKLDITSHNEDYTNVEQYERAEGRHSTGA

>09
MASSEDVIKEFMRFKVCMKGSVNGHVFEIEGEGEGRPYEGTQSVKLKVTKGGPLPFAWDILSPQF
QYGSKAYVKHPANIPDYVKLSFPEGLKWVRIMNFEDGGVVTVTHDSSLQDGEFIYKVRLVGIDFP
SDGPVMQKRTMGWEASTERMYPEDGALKGSVKMRLKLKDGGHYNAEVNTTYMAKKPVQLPGAYIT
DIKLDITSHNDDYTVVEQYERAEGRHSTGA

>10
MASSEDVIKEFMRFKVSMEGSVNGHEFEIEGVGEGRPYEGTQSAKLKVTKGGPLPFAWDILSPQF
QYGSKVYVKHPADIPDYLKLSFPEGFKWIRIMNFEDGGVVTVTQDSSLQDGVFIYKVKLHGIDFP
SDGPVMQKQTMGWEASTERMYPEDGALQGAVKMRLKLKDGGRYGAEVKTTYMAKKHVQLPGAYLT
DIKLDITSHNDDYTIVEQYERAEGRHSTGA

>11
MASSEDVIKDFMRFRVRMEGSVNGHEFEIEGEGEGHPYEGTQSAKLKVTKGGPLPFAWDILSPQF
MWGSKAYVKHPVDIPDYMKLSFPEGFKWERVMNFEDGGVVIVTQDSSLQDGEFVYEVRLCGTNFP
SDGPVMQKKTMGCAAFSERIYSEDGALKGEVKMRLRLKDGDHYEAEVNTTYKAKKAVQLPDAYII
YGKLDIISHNEDYTIVEQYERAEGRHSTGA

>12
MASSEDVIKEFMRFKVRMEGSVNGHEFEIEGEGAGRPYEGTQAAKLKVIKGGPLPFAWDILSPQL
MWGSKAYVKHPADIPDYMKLSFPEGFKWERVMIFEDGGVLTVTQDSSLQDGEFIYKVRLCGTNFP
SDGPVMQKRTMGCAAISERTYPEDGALKGEVKMRLRLKDGGHYEAEVKTTYMAKKTVQLPDAYII
DGKLDIISHNEDYTIVEQYERAEGRHSTGA

>13
MASSEDVIKEFMRFKVRMVGSVNGHEFEIEGEGEGRPYEGTQAAKLKVTKGGPLPFAWDILSPQF
MWGSKAYVKHPADIPDYMKLSFPEGFRWERVMIFEDGGVVTVTQDSSLLDGEFIYEVKLCGTNFP
SDGPVMQKKTMGCAAISERIYPKDGALKGEVVMRLRLKDGDHYVAEVKTTYTAKQAVPLPDAYII
DGKLDIISYNEDYTIVEQYERAEGRHSTGA

>14
MASSEDVIKEFMRFKVRMEGSVNGHEFEIEGEGEGRPYEGTQTAKLKVTKGGPLPFSWDILSPQF
MWGSKAYVKHPADIPDYMKLSFPEGFKWDRVMIFEDGGVVTVTQDSSLQDGVLIYKVKVRGTNFP
SDGPVMQKKTMGCAAISERVYPEDGALKGRVKMRLRLKGDDYYDAEVSTTYMAKKLVQLPDAYNI
DGKLDIISHNKDYTIVEQYERAEARHSTGV

>15
MASSEDVIKEFMRFKVRMEGSVNGHEFEIEGEGEGRPYEGTQTAKLKVTKGGPLPFSWDILSPQF
MWGSKAYVKHPADIPDYMKLSFPEGFKWDRVMNFEDGGVVIVTHDSSLQDGVFIYKVKLCGTNFP
SVGPVMQKRTMGCAAISERIYPEDGALKGEVKMRLRLKDGGHYGAEVNTTYMAKKTVQLPGAYEI
DGKLDIISHNEDYTIVEQYERAEGRHSTGA

>16
MASSEDVIKEFMRYKVRMVGSVNGHEFEIKGEGEGRPYEGTQTAILKVTKGGPLPFSWDILSPQF
MWGSKAYVKHSADIPDYMKLSFPEGFKWYRVMNFEDGGVITVTHDSSLHDGEFINEVKLRGTNFP
SDGPVMQKKTKGCAAFSEHIYPEDGALKGDVIMRLRLEDGDHYVAEVSTAYIAKKRVQLPDAYKI
DGKLDIISHNEDYTIVEQYERAEGRHSIGA

>17
MASSEDVIKEFMRYKVRMEGSVNGHEFEIEGEGKGRPYEGTQTAKLKVTKGGPLPFSWDILSPQF
MWGSKAYVKHPADIPDYMKLSFPEGFKWYRVMNFEDGGVVTVTQDSSLQDGKFIYEVKLRGINFP
SDGPVMQKKTMGCAAFSELTYPEDGALKGDVIMRLRLKDGRHYNAEVSTTYMAKKSVQLPDAYII
DGKLDIISHNEDYTIVEQYERAEGRHFTGA

>18
MASSEDVIKEFMRYKVRMEGSVNGHEFEIEGVGEGRPYEGTQTAKLSVTKGGPLPFSWDILSPQF
MWGSKTYVKHPPDIPDYMKQSFPEGFRWYRVMNFEDGGVITVTQDSSLQDGKFTYEVKLHGTNFP
SHGPVMQKKTNGYAAFSERIYPVDGALKGDVIMRLRLKDGNHYDAQVSTIYMAKKTVQLPDEYKI
NGKLDITSHNEDYTIVEQYERAEGRHSTGA

>19
MASSEDVIKEFMRYKVRMEGSINGHEFEIEGVGEGRPYEGTQTAKLRVTRGGPLPFSWDILSPQF
MWGSKTYVKHPSDIPDYMKLSFPEGFRWYRVMNFEDGGVITVTQDSSLKDGEFIYEVKLHGTNFP
SYGPVMQKKTIGYAAFSERMYPVDGALKGDVIMRLRLKDGSHYDAEVRTTYMAKKTVQLPEEYKI
DGKLDITSHNEDYTIVEQYERAEGRHSTGA

>21
MASSEDVIKEFMRFKVRMEGSVNGHEFEIEGEGEGRPYEGTQAAKLKVTKGGPLPFAWDILSPQF
QYGSKAYVKHPADIPDYMKLSFPEGFKWDRVMNFEDGGVVTVTQDSSLQDGEFIYKVKLRGTNFP
SDGPVMQKKTMGWEASTERMYPEDGALKGEVKMRLKLKDGDHYDAEVNTTYMAKKPVQLPGAYKT
DVKLDITSHNEDYTIVEQYERAEGRHSTGA

>22
MASSEDVIKEFMRFKVRMEGSVNGHEFEIEGEGEGRPYEGTQAAKLKVTKGGPLPFAWDILSPQF
MWGSKAYVKHPADIPDYMKLSFPEGFKWDRVMNFEDGGVVTVTQDSSLQDGEFIYEVKLRGTNFP
SDGPVMQKKTMGCAAISERIYSEDGALKGEVKMRLRLKDGDHYDAEVNTTYMAKKTVQLPDAYKI
DGKLDIISHNEDYTIVEQYERAEGRHSTGA

>23
MASSEDVIKEFMRFKVRMEGSVNGHEFEIEGEGEGRPYEGTQTAKLKVTKGGPLPFSWDILSPQF
MWGSKAYVKHPADIPDYMKLSFPEGFKWDRVMNFEDGGVVTVTQDSSLQDGEFIYEVKLRGTNFP
SDGPVMQKKTMGCAAISERIYPEDGALKGEVKMRLRLKDGDHYDAEVSTTYMAKKTVQLPDAYKI
DGKLDIISHNEDYTIVEQYERAEGRHSTGA

>24
MASSEDVIKEFMRYKVRMEGSVNGHEFEIEGEGEGRPYEGTQTAKLKVTKGGPLPFSWDILSPQF
MWGSKAYVKHPADIPDYMKLSFPEGFKWYRVMNFEDGGVVTVTQDSSLQDGEFIYEVKLRGTNFP
SDGPVMQKKTMGCAAFSERIYPEDGALKGDVIMRLRLKDGDHYDAEVSTTYMAKKTVQLPDAYKI
DGKLDITSHNEDYTIVEQYERAEGRHSTGA

>25
MASSEDVIKEFMRYKVRMEGSVNGHEFEIEGEGEGRPYEGTQTAKLRVTKGGPLPFSWDILSPQF
MWGSKTYVKHPADIPDYMKLSFPEGFRWYRVMNFEDGGVITVTQDSSLQDGKFIYEVKLRGTNFP
SYGPVMQKKTIGYAAFSERMYPEDGALKGDVIMRLRLKDGDHYDAEVSTTYMAKKTVQLPDEYKI
DGKLDITSHNEDYTIVEQYERAEGRHSTGA

>26
MASSEDVIKEFMRYKVRMEGSVNGHEFEIEGEGEGRPYEGTQTAKLKVTKGGPLPFSWDILSPQF
MWGSKAYVKHPADIPDYMKLSFPEGFKWYRVMNFEDGGVVTVTQDSSLQDGEFIYEVKLRGTNFP
SDGPVMQKKTMGCAAFSERIYPEDGALKGDVIMRLRLKDGDHYDAEVSTTYMAKKTVQLPDAYKI
DGKLDIISHNEDYTIVEQYERAEGRHSTGA

>27
MASSEDVIKEFMRFKVRMEGSVNGHEFEIEGEGEGRPYEGTQTAKLKVTKGGPLPFSWDILSPQF
MWGSKAYVKHPADIPDYMKLSFPEGFKWDRVMNFEDGGVVTVTQDSSLQDGVFIYEVKLRGTNFP
SDGPVMQKKTMGCAAISERIYPEDGALKGEVKMRLRLKDGDHYDAEVSTTYMAKKTVQLPDAYKI
DGKLDIISHNEDYTIVEQYERAEGRHSTGA

>28
MASSEDVIKEFMRFKVRMEGSVNGHEFEIEGEGEGRPYEGTQAAKLKVTKGGPLPFAWDILSPQF
MWGSKAYVKHPADIPDYMKLSFPEGFKWERVMNFEDGGVVTVTQDSSLQDGEFIYEVKLCGTNFP
SDGPVMQKKTMGCAAISERIYSEDGALKGEVKMRLRLKDGDHYDAEVNTTYMAKKTVQLPDAYII
DGKLDIISHNEDYTIVEQYERAEGRHSTGA

>29
MASSEDVIKEFMRFKVRMEGSVNGHEFEIEGEGEGRPYEGTQAAKLKVTKGGPLPFAWDILSPQF
MWGSKAYVKHPADIPDYMKLSFPEGFKWERVMIFEDGGVVTVTQDSSLQDGEFIYEVKLCGTNFP
SDGPVMQKKTMGCAAISERIYPEDGALKGEVKMRLRLKDGDHYEAEVKTTYMAKKTVQLPDAYII
DGKLDIISHNEDYTIVEQYERAEGRHSTGA

>30
MASSEDVIKEFMRFKVCMEGSVNGHEFEIEGEGEGRPYEGTQAAKLKVTKGGPLPFAWDILSPQF
QYGSKAYVKHPADIPDYMKLSFPEGFKWDRVMNFEDGGVVTVTQDSSLQDGEFIYKVKLRGTDFP
SDGPVMQKQTMGWEASTERMYPEDGALKGGVKMRLKLKDGGHYDAEVNTTYMAKKPVQLPGAYIT
DIKLDITSHNEDYTIVEQYERAEGRHSTGA

>31
MASSEDVIKEFMRFKVCMEGSVNGHEFEIEGEGEGRPYEGTQSAKLKVTKGGPLPFAWDILSPQF
QYGSKVYVKHPADIPDYVKLSFPEGFKWVRIMNFEDGGVVTVTQDSSLQDGEFIYKVKLHGIDFP
SDGPVMQKQTMGWEASTERMYPEDGALKGAVKMRLKLKDGGHYDAEVNTTYMAKKPVQLPGAYIT
DIKLDITSHNDDYTIVEQYERAEGRHSTGA

>32
MASSEDVIKEFMRFKVCMEGSVNGHEFEIEGEGEGRPYEGTQAAKLKVTKGGPLPFAWDILSPQF
QYGSKAYVKHPADIPDYMKLSFPEGFKWDRVMNFEDGGVVTVTQDSSLQDGEFIYKVKLRGTDFP
SDGPVMQKQTMGWEASTERMYPEDGALKGEVKMRLKLKDGGHYDAEVKTTYMAKKPVQLPGAYIT
DIKLDITSHNEDYTIVEQYERAEGRHSTGA

>33
MASSEDVIKEFMRFKVCMEGSVNGHEFEIEGEGEGRPYEGTQAAKLKVTKGGPLPFAWDILSPQF
QYGSKAYVKHPADIPDYMKLSFPEGFKWDRVMKFEDGGVVTVTQDSSLQDGVFIYKVKLRGTDFP
SDGPVMQKQTMGWEASIERMYPEDGLLKGEAKMRLKLKNGGHYDAEVKTTYMAKKPVQLPGAYIT
DIKLDITSHNEDYTIVEQYERAEGRHSTGA

>34
MASSEDVIKEFMRFKVYLEGSVNGHEFEIEGEGEGRPYEGTQVAKLKVTKGGPLPFAWDILSPQF
QYGSKAYVKHPADIPDYMKLSFPEGFKWDRVMNFEDGGVVTVTQDTSLQDGEFICKVKLRGTDFP
SEGPVMQKQTMGWEASTERMYPEDGALKGEDKMRLRLKDGGHYDADVKTTYMAKKPVQLPGAYIT
DIKLDITSHNEDYTIVEQYERAEGRHSTGA

>35
MASSEDVIKEFMRFKVYLEGSVNGHEFEIEGEGEGRPYEGTQVAKLKVTKGGPLPFAWDILSPQF
QYGSKAYVKHPADIPDYMKLSFPEGFKWDRVMNFEDGGVVTVTQDTSLQDGEFIYKVKLRGTDFP
SEGPVMQKQTMGWEASTERMYPEDGALKGEDKMRLRLKDGGHYEADVKTTYMAKKPVQLPGAYIT
DIKLDITSHNEDYTIVEQYERAEGRHSTGA

>36
MASSEDVIKEFMRFKVYLEGSVNGHEFEIEGEGEGRPYEGTQVAKLRVTKGGPLPFAWDILSPQF
QYGSKAYVKHPADIPDYMKLSFPEGFKWDRVMNFEDGGVVTVTQDTSLQDGEFICKVKLRGTDFP
SEGPVMQKQTMGWEASTERMYPEDGVLKGEDKMRLRLKDGGHYDAYVKTTYMAKKPVQLPGAYIT
DIKLDVTSHNEDYTIVEQYERAEGRHSTGA

>37
MASSEDVIKEFMRFKVSMEGSVNGHEFEIEGEGEGRPYEGTQTAKLKVTKGGPLPFAWDILSPQF
QYGSKAYVKHPADIPDYLKLSFPEGFKWERVMNFEDGGVVTVTQDSTLQDGVLIYKVKLRGTNFP
SDGPVMQKKTRGWEASTERMYPEDGVLKGEIKMRLKLKDGSHYEAEVKTTYMAKKPVQLPGAYKT
DIKLDITSHNEDYTIVEQYERAEGRHSTGA
